# Supplementary material for: Association of the rs9896052 Polymorphism Upstream of GRB2 with Proliferative Diabetic Retinopathy in Patients with Less than 10 Years of Diabetes
Source: Int J Mol Sci. 2024 Sep 24;25(19):10232. doi: 10.3390/ijms251910232 (PMC11477274; doi:10.3390/ijms251910232)
Supplement: Supplementary file 1 [file ijms-25-10232-s001.zip › ijms-3144399-supplementary.pdf]

Supplementary Table S1. Univariate and backward stepwise multiple logistic regression analysis of the association between the rs9896052 polymorphism and PDR in the overall group of patients with T2DM.

Variable	Unadjusted OR (95% CI)	Adjusted OR (95% CI)
CC genotype *	1.18 (0.86–1.62)	0.90 (0.58–1.39)
Male gender	2.37 (1.77–3.18)	1.60 (1.08–2.36)
T2DM duration (years)	1.08 (1.06–1.10)	1.05 (1.03–1.08)
Daily insulin use (yes/no)	2.81 (2.08–3.80)	2.02 (1.35–3.02)
Body mass index (kg/m²)	0.92 (0.89–0.95)	0.95 (0.91–0.98)
eGFR (mL/min/1.73m²)	0.97 (0.97–0.98)	0.97 (0.97–0.98)
Active smoking (yes/no)	0.50 (0.30–0.82)	0.84 (0.40–1.77) **
Age at T2DM diagnosis (years)	0.98 (0.97–1.00)	1.02 (0.99–1.05) ***
Systolic blood pressure (mmHg)	1.01 (1.00–1.01)	1.01 (1.00–1.02) ****
Non-white skin color	0.96 (0.68–1.35)	1.63 (0.86–3.09) *****
Glycated hemoglobin (%)	0.91 (0.84–0.99)	0.93 (0.82–1.04) ******

Abbreviations: PDR—proliferative diabetic retinopathy; T2DM—type 2 diabetes mellitus; OR—odds ratio; CI—confidence interval; eGFR—estimated glomerular filtration rate. *—versus AC + AA genotypes; **—removed in step 2; ***—removed in step 3; ****—removed in step 4; *****—removed in step 5; ******—removed in step 6.

Supplementary Table S2. Clinical, laboratory, and demographic characteristics of patients with T2DM according to their rs9896052 genotypes.

Variable	AA (*n* = 188)	AC (*n* = 415)	CC (*n* = 235)	*p* Value
Age (years)	58.9 ± 9.6	60.0 ± 9.3	60.9 ± 9.6	0.103
Male gender	79 (42.0%)	181 (43.6%)	115 (48.9%)	0.294
White	111 (59.0%)[a]	336 (81.0%)[b]	204 (86.8%)[b]	<0.001
Age at T2DM diagnosis (years)	46.2 ± 9.3	46.7 ± 9.3	47.6 ± 9.6	0.227
T2DM duration (years)	12.7 ± 7.4	13.3 ± 8.0	13.3 ± 8.4	0.817
Glycated hemoglobin (%)	8.0 ± 2.2[a]	7.6 ± 2.2[a,b]	7.3 ± 1.9[b]	0.024
Daily insulin use	47.3%	46.3%	46.8%	0.976
Body mass index (kg/m^2)	29.8 ± 5.6	29.7 ± 5.9	30.0 ± 6.3	0.828
Smoking				0.389
Never smoker	51.6%	55.2%	53.1%	
Former smoker	31.7%	33.8%	35.1%	
Active smoker	16.7%	10.9%	11.8%	
Hypertension	78.0%	74.9%	75.0%	0.715
Systolic BP (mmHg)	141.6 ± 22.2	141.8 ± 23.1	141.9 ± 24.4	0.990
Diastolic BP (mmHg)	83.9 ± 11.1	84.3 ± 13.6	84.0 ± 14.7	0.598
eGFR (mL/min/1.73m^2)	94 (64–111)[a]	91 (62–105)[a,b]	87 (55–101)[b]	0.045
Diabetic kidney disease	61.2%	56.8%	64.3%	0.238
Total cholesterol (mmol/L)	5.08 ± 1.44	5.18 ± 1.21	5.00 ± 1.28	0.267
HDL cholesterol (mmol/L)	1.09 (0.87–1.34)	1.09 (0.88–1.34)	1.10 (0.93–1.29)	0.987
LDL cholesterol (mmol/L)	2.78 (2.19–3.92)	2.97 (2.35–3.88)	2.88 (2.12–3.78)	0.387
Triglycerides (mmol/L)	1.84 (1.17–3.01)	1.76 (1.33–2.63)	1.78 (1.28–2.69)	0.888

Abbreviations: T2DM—type 2 diabetes mellitus; BP—blood pressure; eGFR—estimated glomerular filtration rate. Data are expressed as mean ± standard deviation, median (25th–75th percentiles), number of individuals (percentage), or percentage. The superscript letters indicate whether the means, medians, or percentages showed statistically significant differences between groups after the correction for multiple pairwise comparisons. Means, medians, or percentages indicated with the same superscript letter did not differ significantly ($p > 0.05$), while the means, medians, or percentages indicated with different superscript letters were significantly different ($p < 0.05$).

Supplementary Table S3. Univariate and backward stepwise multiple logistic regression analysis of the association between the rs9896052 polymorphism and PDR in white patients with T2DM.

Variable	Unadjusted OR (95% CI)	Adjusted OR (95% CI)
CC genotype *	1.34 (0.95–1.90)	1.06 (0.67–1.66)
Male gender	2.39 (1.71–3.33)	1.83 (1.20–2.79)
T2DM duration (years)	1.08 (1.06–1.10)	1.04 (1.02–1.07)
Daily insulin use (yes/no)	3.14 (2.22–4.42)	2.38 (1.55–3.65)
eGFR (mL/min/1.73m^2)	0.97 (0.96–0.98)	0.98 (0.97–0.98)
Age at T2DM diagnosis (years)	0.98 (0.96–1.00)	1.02 (0.99–1.05) **
Glycated hemoglobin (%)	0.90 (0.82–1.00)	0.90 (0.79–1.03) ***
Body mass index (kg/m^2)	0.93 (0.90–0.96)	0.96 (0.92–1.01) ****

Abbreviations: PDR—proliferative diabetic retinopathy; T2DM—type 2 diabetes mellitus; OR—odds ratio; CI—confidence interval; eGFR—estimated glomerular filtration rate. *—versus AC + AA genotypes; **—removed in step 2; ***—removed in step 3; ****—removed in step 4.

Supplementary Table S4. Univariate and backward stepwise multiple logistic regression analysis of the association between the rs9896052 polymorphism and PDR in non-white patients with T2DM.

Variable	Unadjusted OR (95% CI)	Adjusted OR (95% CI)
CC genotype *	0.54 (0.22–1.32)	1.07 (0.37–3.13)
T2DM duration (years)	1.08 (1.04–1.14)	1.05 (1.00–1.11)
Body mass index (kg/m^2)	0.90 (0.84–0.95)	0.92 (0.86–0.99)
eGFR (mL/min/1.73m^2)	0.97 (0.96–0.98)	0.98 (0.97–0.99)
Age (years)	1.04 (1.00–1.07)	1.01 (0.95–1.07) **
Daily insulin use (yes/no)	2.00 (1.06–3.78)	1.11 (0.42–2.91) ***
Male gender	2.35 (1.26–4.40)	1.21 (0.46–3.18) ****
Having a mother with diabetes (yes/no)	2.02 (1.03–3.96)	1.97 (0.79–4.95) *****
Active or former smoking (yes/no)	0.45 (0.23–0.87)	0.60 (0.27–1.34) ******

Abbreviations: PDR—proliferative diabetic retinopathy; T2DM—type 2 diabetes mellitus; OR—odds ratio; CI—confidence interval; eGFR—estimated glomerular filtration rate. *—versus AC + AA genotypes; **—removed in step 2; ***—removed in step 3; ****—removed in step 4; *****—removed in step 5; ******—removed in step 6.

Supplementary Table S5. Univariate and backward stepwise multiple logistic regression analysis of the association between the rs9896052 polymorphism and PDR in those with a long duration of T2DM (≥10 years).

Variable	Unadjusted OR (95% CI)	Adjusted OR (95% CI)
CC genotype *	1.00 (0.68–1.47)	0.73 (0.42–1.25)
T2DM duration (years)	1.09 (1.06–1.12)	1.06 (1.02–1.10)
Daily insulin use (yes/no)	3.12 (2.17–4.49)	2.55 (1.58–4.13)
Body mass index (kg/m²)	0.95 (0.92–0.98)	0.96 (0.91–1.00)
eGFR (mL/min/1.73m²)	0.97 (0.96–0.98)	0.97 (0.96–0.98)
Age at T2DM diagnosis (years)	0.98 (0.96–1.00)	1.00 (0.97–1.03) **
Active smoking (yes/no)	0.53 (0.30–0.95)	0.96 (0.43–2.14) ***
Hypertension (yes/no)	1.65 (1.03–2.63)	0.79 (0.42–1.47) ****
Male gender	1.97 (1.40–2.78)	1.38 (0.86–2.20) *****
Non-white skin color	1.01 (0.67–1.51)	1.67 (0.94–2.95) ******

Abbreviations: PDR—proliferative diabetic retinopathy; T2DM—type 2 diabetes mellitus; OR—odds ratio; CI—confidence interval; eGFR—estimated glomerular filtration rate. *—versus AC + AA genotypes; **—removed in step 2; ***—removed in step 3; ****—removed in step 4; *****—removed in step 5; ******—removed in step 6.

Supplementary Table S6. Clinical, laboratory, and demographic characteristics of patients with T2DM according to the known duration of their diabetes.

Variable	< 10 years (*n* = 281)	≥ 10 years (*n* = 557)	*p* Value
Age (years)	55.9 ± 9.9	62.0 ± 8.6	<0.001
Male gender	115 (40.9%)	260 (46.7%)	0.132
White	218 (77.6%)	433 (77.7%)	>0.999
Age at T2DM diagnosis (years)	50.8 ± 9.7	44.8 ± 8.5	<0.001
T2DM duration (years)	5.1 ± 2.4	17.2 ± 6.6	<0.001
Glycated hemoglobin (%)	7.5 ± 2.2	7.6 ± 2.1	0.259
Daily insulin use	33.7%	53.0%	<0.001
Body mass index (kg/m^2)	31.2 ± 6.6	29.1 ± 5.4	<0.001
Smoking			0.413
Never smoker	51.4%	55.0%	
Former smoker	34.1%	33.5%	
Active smoker	14.5%	11.5%	
Hypertension	66.7%	80.2%	<0.001
Systolic blood pressure (mmHg)	138.2 ± 23.2	143.6 ± 23.1	<0.001
Diastolic blood pressure (mmHg)	84.5 ± 13.2	84.0 ± 13.5	0.840
eGFR (mL/min/1.73m^2)	98 (73–111)	85 (54–102)	<0.001
Diabetic kidney disease	65.5%	57.4%	0.063
Total cholesterol (mmol/L)	5.17 ± 1.28	5.07 ± 1.29	0.147
HDL cholesterol (mmol/L)	1.03 (0.85–1.27)	1.11 (0.93–1.34)	0.001
LDL cholesterol (mmol/L)	3.10 (2.26–3.94)	2.87 (2.28–3.80)	0.324
Triglycerides (mmol/L)	1.98 (1.40–3.01)	1.72 (1.21–2.45)	0.001
Proliferative diabetic retinopathy	60 (21.4%)	224 (40.2%)	<0.001

Abbreviations: T2DM—type 2 diabetes mellitus; eGFR—estimated glomerular filtration rate. Data are expressed as mean ± standard deviation, median (25th–75th percentiles), number of individuals (percentage), or percentage.
